# Can Pre-operative MRI Predict the Need for Salter’s Osteotomy in DDH Children Undergoing Open Reduction?

**DOI:** 10.5704/MOJ.2503.010

**Published:** 2025-03

**Authors:** KC Vatsyan, K Rangasamy, NR Gopinathan, P Sudesh, A Shinha, AK Salaria

**Affiliations:** 1 Department of Orthopaedics, Shri Lal Bahadur Shastri Government Medical College and Hospital, Mandi, India; 2 Department of Orthopaedics, Post Graduate Institute of Medical Education and Research (PGIMER), Chandigarh, India; 3 Department of Radiology, Post Graduate Institute of Medical Education and Research (PGIMER), Chandigarh, India; 4 Department of Orthopaedics, All India Institute of Medical Sciences (AIIMS), Bilaspur, India

**Keywords:** DDH, Salter's osteotomy, acetabular index, open reduction, intra-operative stability test

## Abstract

**Introduction:**

MRI having the multiplanar capability is a good choice for pre-operative planning in developmental dysplasia of hip (DDH). Although few previous studies utilised MRI to quantify dysplasia, predict outcomes, and the procedure required, there are no defined pre-operative conclusive criteria on when to do Salter's osteotomy?

**Material and Methods:**

A prospective cohort study was conducted in unilateral idiopathic DDH cases those who underwent an open reduction in the age group of one to four years. Pre- and post-operative MRI was done to assess various acetabular and femoral parameters. Intra-operatively, osteotomy was planned. Based on stability assessment given by Zadeh *et al* Clinical follow-up assessment was done at three- and six-month post-op. Functional assessment using Modified McKay's criteria was done at six months follow-up.

**Results:**

Out of 15 cases, seven children underwent only open reduction (OR), whereas eight underwent OR with Salter's osteotomy. Based on pre-op acetabular index and anteversion, Salter's osteotomy should be done in 14 out of 15 cases, but intra-operative stability test precluded Salter's in 6 cases. Post-operative anterior sectoral angle and femoral head coverage percentage were better in OR with Salter's group than OR-only group, but not statistically significant. Functional assessment at final follow-up showed all OR with Salter's group cases were Grade I, whereas in OR-only group, 4 were Grade I and 3 were Grade II.

**Conclusion:**

Three-dimensional dynamic assessment using intra-operative stability test predicts the best possible interrelation between the articular surface of the femoral head and acetabulum and the need for osteotomy rather than preoperative MRI.

## Introduction

Developmental dysplasia of the hip (DDH) refers to a spectrum of hip disorders that range in severity from mild acetabular dysplasia to severe unstable and dislocated hip^1^. The incidence of hip instability in the newborn has been reported between 5 and 13 per 1000 births. About 90% of cases with mild hip instability at birth resolve spontaneously within the first eight weeks of life^2^. Diagnosis is usually made clinically and confirmed with various radiological modalities like Ultrasonography (USG), radiographs (radiograph imaging), Computed tomography (CT) scanning, and Magnetic resonance imaging (MRI)^3,4^.

Several radiographic parameters have been defined quantifying the amount of dysplasia and femoral head dislocation/subluxation in DDH like acetabular index, centre edge (CE) angle of Wiberg, centre of the superior margin of metaphysis relation to Hilgenreiner's (H) line, Perkin's (P) lines, and diagonal (D) line, break-in Shenton's line, and delay in appearance of ossification centre^1^.

It is true radiograph is a traditional modality to evaluate DDH, but its role is quite limited due to 2D Imaging. Multiplanar imaging and 3D reconstruction (CT and MRI) may be most helpful to clinicians in determining the planning for roof osteotomies than normal radiograph.

With the advent of MRI and its increasing application, more details regarding the non-ossified cartilage and soft tissues in DDH are now available. MRI is a good choice for preoperative planning because of the absence of ionizing radiation, the better visualisation of soft tissue structures, and the multiplanar capability^5^.

Few studies in the literature have attempted to quantify the femoral and acetabular parameters by MRI and correlate them with clinical and functional outcomes^6,7^. Also, the utility of anterior and posterior sectoral angles in prognosticating these children's results is not very well defined. Post-operative assessment and quality of reduction were also studied previously^8^. But there are no conclusive criteria to find pre-operatively which child requires additional Salter’s osteotomy to maintain a stable reduction.

Our primary objective of the study was to evaluate the preoperative predictive accuracy of MRI for OR (open reduction) with or without Salter's osteotomy, and secondary objectives were to assess acetabular and femoral parameters and comparison with normal side and the post-operative assessment of the adequacy of reduction. We hypothesised that pre-operative MRI parameters would help decide which DDH child requires Salter's osteotomy to maintain a stable and concentric reduction.

## Materials and Methods

This prospective cohort study was conducted on children presenting to the Paediatric Orthopaedic Clinic of a tertiary care Centre for a period of one year (July 2016 to June 2017). Children between the age of 1 and 4 years of either gender diagnosed with unilateral idiopathic DDH based on clinical and radiological examination and whose parents/guardians were consenting to participate in the study were included. Only those children who required open reduction with or without Salter's innominate osteotomy or femoral derotational/varus osteotomy were included. The surgical plan was made after assessment and mutual agreement between senior consultants (PS, NRG). Institute Ethics committee approval was obtained (NK/3116/MS/17197-98) before enrolling the participants.

Children with a history of neonatal hip septic arthritis or significant trauma to the hip, syndromic (like arthrogryposis) DDH, neurogenic DDH, bilateral DDH, those children managed by closed reduction and spica casting, and children who underwent acetabuloplasty surgeries were excluded.

A detailed assessment of the enrolled children was done by history-taking, physical examination, and radiological evaluation. MRI in children one to four years required sedation. Under the supervision of department of Anaesthesia in all of our cases MRI was performed under mild sedation (Syrup Triclofos 50mg/kg), no additive anaesthesia was required.

Pre-operative and post-operative non-contrast MRI of bilateral hips was done on a three Tesla MR machine [Magnetom Verio, Siemens, Erlangen, Germany] under sedation for all participants. T1-weighted spin-echo (TR 500-730 ms - TE- 19-23ms, slice thickness 3mm) and T2-weighted fat-saturated turbo spin-echo (TR-3700 -5860 ms TE-76ms, slice thickness-3mm) coronal and axial sequences were acquired. To calculate femoral anteversion, axial T2-weighted sections through the knee joint were taken. Both the lower limbs were kept in a symmetrical position throughout the scanning. The following parameters were measured bilaterally.

**Fig. 1:**
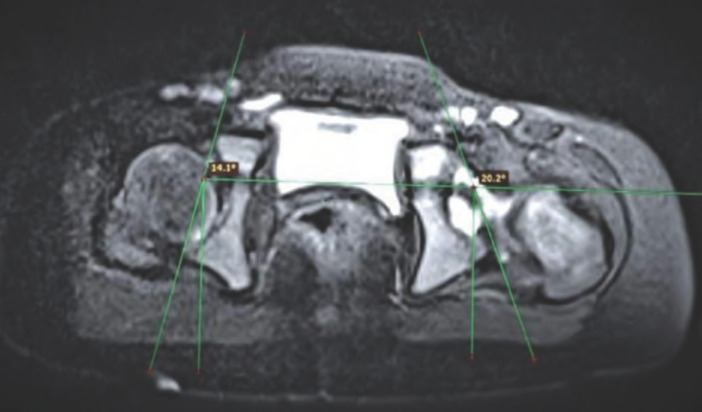
Acetabular anteversion (AA) angle: Axial T2-weighted fat-suppressed image of the hip in a patient with left sided DDH. The AA angle is 14.1° on the right side and increased (20.2°) on the left side.

Acetabular Anteversion was measured by drawing a reference line perpendicular to the transradiate line. The bony acetabular axis across the margins from posterior edge to anterior edge was drawn. The angle of acetabular anteversion was calculated as the angle formed between these two axes^9^ (Fig. 1).

**Fig. 2:**
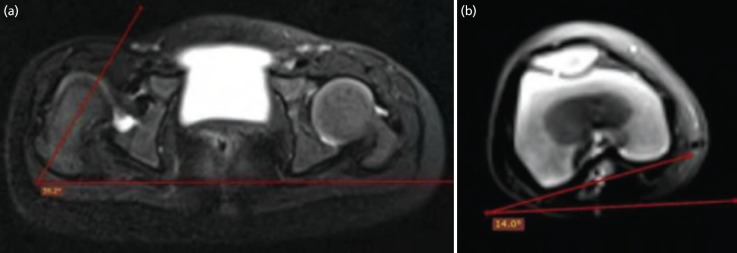
Femoral anteversion (FA) angle: Axial T2-weighted fat-suppressed image of the hip with right-sided DDH in a 1-year-old female child. (a) The α angle (59.2°) is the angle between the line parallel to femoral neck and the horizontal reference line. (b) The ß angle (14°) is between the line through the posterior ipsilateral femoral condyles and the horizontal reference line. The FA angle is corrected (α -ß) and it is calculated as 45.2°.

For Femoral Anteversion Oblique, axial, and sagittal sections placed parallel to the femoral neck axis and perpendicular to the table were taken. An α angle between the neck axis and horizontal reference line was calculated. The β angle between the posterior tangent and the horizontal reference line was determined on axial sections through the centre of the femoral condyles. Depending upon the rotation of the femur, the femoral anteversion can be calculated by α±β. It was made sure during the entire procedure that the patient should not move, so that proximal and distal femoral cuts are taken in the same rotational status^10^ (Fig. 2).

**Fig. 3:**
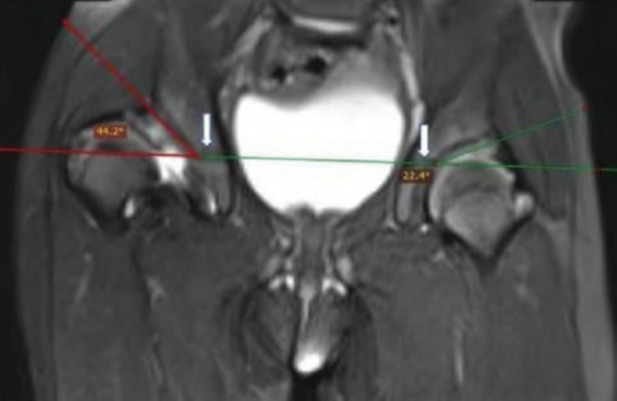
Cartilaginous Acetabular index (CAI): Coronal T2-weighted fat-suppressed image of the hip with right-sided DDH in a 1-year-old female child. The acetabular index is calculated by an angle formed by a line passing through superior aspect of triradiate cartilage (arrows) i.e., Hilgenreiner’s line, and a second line along the acetabular roof. The right acetabular index (44.2°) is increased as compared to the normal left side (22.4°).

Cartilaginous acetabulum should be taken into account in Acetabulum Index (AI) measurements. MRI may be a more appropriate option for the evaluation of acetabular cartilaginous coverage in the evaluation of AI and the decision to perform surgery. Cartilaginous Acetabulum Index (CAI) calculated by measuring the angle between a horizontal line connecting both superior aspects of triradiate cartilages (Hilgenreiner's line) and a second line that extends along the acetabular roof^11^ (Fig. 3).

**Fig. 4:**
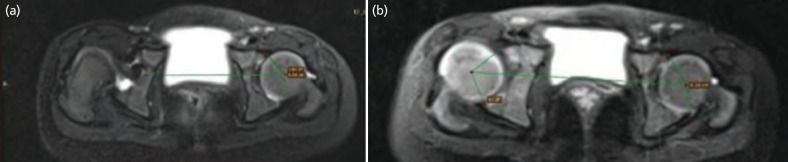
Anterior and Posterior acetabular sector angles (ASA and PSA), (a) T2-weighted fat-saturated image of the hip in a 2-year-old female had right DDH. (b) The same child underwent OR with Salter’s osteotomy for right DDH. The anterior and the posterior sector angles on the right side are 40.1° and 67.8°.

Anterior and Posterior sectoral angles are determined by measurement of the angle drawn between the centre of the femoral head and the anterior and posterior acetabular rim relative to the horizontal axis of the pelvis (Fig. 4).

For femoral head coverage, we utilised the Remier’s index on MRI. MRI can give better femoral head coverage as role of cartilaginous parts also included. In all cases we took section where maximum cartilaginous head coverage was present.

**Fig. 5:**
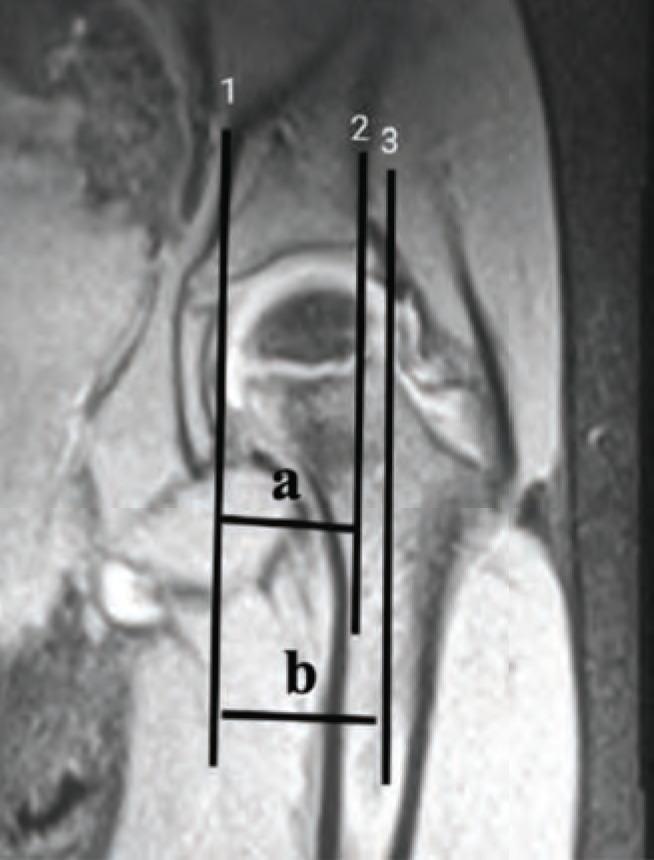
Percentage of femoral head coverage (PFHC): The 3 lines used to calculate the PFHC are line 1 drawn through medial aspect of acetabulum, line 2 through lateral acetabular rim, and line 3 through lateral aspect of femoral head. (a) The PFHC is calculated as the ratio of distance between line 1 and 2 / (b) distance between line 1 and 3.


Three vertical lines are utilised to determine percentage of Femoral Head coverage. Line 1 passing perpendicular through the medial most aspect of the hip joint, Line 2 passing through the lateral part of the acetabulum, and line 3 passing through the lateral most of the femoral head were calculated^12^ (Fig. 5).

$$\text{Percentage}\text{acetabulum}\text{coverage}=\frac{\text{Distance}\text{between}1\text{and}2\left[\text{a}\right]\times 100}{\text{Distance}\text{between}1\text{and}3\left[\text{b}\right]}$$

Acetabular coverage of more than 75% was taken as normal. The normal side of the hip was taken as a control in unilateral DDH. Surgery was done under general anaesthesia in all our cases. Among healthy children with a single anaesthesia exposure before age 36 months, compared with healthy siblings with no anaesthesia exposure, there were no statistically significant differences in IQ scores in later childhood^13^. However, some studies shows that long duration GA (>240 minutes) and multiple general anaesthesia exposure can cause neurodevelopmental delay in children^14^. The duration of surgery in all our cases was within 150 minutes.

Anterior open reduction through an oblique bikini incision described by Somerville and with or without femoral derotation osteotomy / Salter's pelvic osteotomy was done under general anaesthesia^15^. All the surgeries were done by a single senior surgeon (NRG) to reduce inter-observer variation.

The need for additional pelvic/femoral osteotomy was decided as per the intra-operative stability described by Zadeh *et al*^16^. Stable reduction is considered to be present when the hip remains reduced with the limb having the axial loading in 30° of flexion, 30° of abduction, and 30° of internal rotation/remain reduced in a neutral position^16^.

Post-operatively, one and a half hip spica was applied for all children, and a post-operative radiograph was done to check reduction. At six weeks, the spica cast was removed, sutures were removed, a radiograph was done to check the adequacy and maintenance of reduction, and an abduction/Petrie cast was applied for another six weeks. After three-month k wires removed under local anaesthesia and clinical and radiological evaluation was done at three months and six months post-operatively to check the stability of the hip and maintenance of reduction, range of motion of the affected hip, any pain, and length discrepancy of limbs. Follow-up MRI was done at six months post-operatively to evaluate femoral and acetabular parameters and adequacy of reduction. Modified McKay's criteria^17,18^ were used for functional assessment at final follow-up of six months postoperatively.

Data were collected in a pre-designed format in an Excel sheet and analysed using Statistical Package for Social Sciences [SPSS Inc Chicago, IL, Version 20.0 for Windows]. For categorical variables, the Chi-square test was applied as the test of association. A total of 95% confidence interval (95% CI) was calculated for each categorical risk factor. Unpaired and paired 't' tests were used for statistical analysis for continuous variables. MRI measurements of normal and diseased hips of study subjects were treated as dependent observations, and therefore unpaired 't' test was used to compare these measurements. Normally distributed data were presented as means and standard deviation with 95% confidence intervals (CI). An association was considered significant if the p-value <0.05. The alpha error was kept at the standard level of 5%, based on which the level of significance was decided and further calculated as shown in the Table I with p values.

**Table I:** Post-operative MRI parameters of affected side comparison between OR-only group with OR plus Salter’s Group with p-values.

Post-operative parameters	Open Reduction only group (in degrees)	Open Reduction with Salter's group (in degrees)	p-values
Femoral Anteversion	29.42±11.80	36.00±6.34	0.22
Acetabulum Anteversion	19.10±12.97	16.82±5.47	0.65
Acetabulum Index	25.52±6.13	20.92±7.86	0.77
Anterior Sectoral Angle	57.28±14.77	59.00±15.34	0.19
Posterior Sectoral Angle	70.42±11.9	66.25±13.49	0.45
Percentage of Femoral Head Coverage	76.28±18.93	81.62±13.42	0.53

## Results

Fifteen cases of idiopathic unilateral DDH that were included in the study were operated during the study period. The mean age at presentation was 30.87±8.13 months. Out of 15 cases of DDH, 8 (53.7%) were right-sided, and seven were left-sided. Only open reduction (OR) was done in 7 (mean age 25±3.51 months) cases and OR with Salter's osteotomy in 8 (mean age 37.14±7.94 months) cases.

The mean pre-op Cartilaginous Acetabular Index (CAI) on the involved side in OR group was found to be 42.57±10.35° and in Salter's group was found to be 43.01±10.29°, while the mean CAI on the normal side in OR group was 21.71±3.54° and in Salter's group was found to be 22.54±4.40°. The mean post-operative CAI on the involved side in OR group was found to be 25.57±6.13° and in Salter's group was found to be 20.92±7.86°, while the overall mean post-operative CAI on the normal side was 19.53±4.57°.

The mean pre-op Acetabular Anteversion (AA) on the involved side in OR group was found to be 24.00±10.40° and in Salter's group was found to be 23.60±5.05°, while the mean AA on the normal side in OR group was 22.71±12.54° and in Salter's group was found to be 22.18±5.34°. The mean post-operative AA on the involved side in OR group was found to be 19.10±12.97° and in Salter's group was found to be 16.82±5.47°, while the overall mean post-operative AA on the normal side was 18.25±12.11°.

The mean pre-op Femoral Anteversion (FA) on the involved side in OR group was found to be 33.14±12.72° and in Salter's group was found to be 37.50±5.88°, while the mean FA on the normal side in OR group was 33.57±9.50° and in Salter's group was found to be 39.25±8.22°. The mean postoperative FA on the involved side in OR group was found to be 29.42±11.80° and in Salter's group was found to be 36.00±6.34°, while the overall mean post-operative FA on the normal side was 32.10±8.06°.

The mean pre-operative ASA/PSA in OR group on the normal side was 58/68.2° and in Salter's group was 56/70°, while the mean pre-operative ASA/PSA on the involved side was not calculated as head was out of acetabulum. The mean post-operative ASA/PSA in OR group on the involved side was 56/81° and in Salter's group was 59/66°.

The mean PFHA in OR group on the normal side was 84.71±7.43° and in Salter's group was 81.00±21.56, involved side PFHA cannot be assessed as the head is out of the acetabulum. The mean post-operative PFHA on the involved side in OR group was 76.28±18.93° and in Salter's group was 81.62±13.42. The various pre-operative MRI acetabular and femoral parameters of the OR-only group and OR with Salters group are shown in Table II and III, respectively.

**Table II:** Pre-operative MRI parameters of OR-only group.

Pre-operative parameters	Control Side (in degrees)	Involved Side (in degrees)
Femoral Anteversion	33.57±9.50	33.14±12.72
Acetabular Anteversion	22.71±12.54	24.00±10.40
Acetabulum Index	21.71±3.54	42.57±10.35
Anterior Sectoral Angle	58.42±7.87	Not Applicable
Posterior Sectoral Angle	68.71±20.25	Not Applicable
Percentage of Femoral Head Coverage	84.71±7.43	Not Applicable

**Table III:** Pre-operative MRI parameters of OR with Salter’s group.

Pre-operative parameters	Control Side (in degrees)	Involved Side (in degrees)
Femoral Anteversion	39.25±8.22	37.50±5.88
Acetabulum Anteversion	22.18±5.34	23.60±5.05
Acetabulum Index	22.54±4.40	43.00±10.29
Anterior Sectoral Angle	56.62±3.583	Not Applicable
Posterior Sectoral Angle	70.87±19.24	Not Applicable
Percentage of Femoral Head Coverage	81.00±21.56	Not Applicable

The analysis shows, pre-op FA (p=0.68), and AA (p=0.62) on the involved and normal side were statistically not significant. The pre-operative AI (p=0.00) on comparison between the involved and normal side shows a statistically significant difference. The mean post-operative AI on OR with Salters group (20.92±7.86) was better than OR-only (25.57±6.13) group but not statistically significant (Table I).

All the 15 hips were operated by Somerville's anterior approach and concentric open reduction was obtained in all the cases. Pre-op MRI parameters (CAI >30°) predicted the need for Salter's osteotomy in 14 out of 15 hips but based on intra-operative stability as described by Zadeh *et al*^16^, only 8 cases underwent open reduction (OR) with Salter's osteotomies, and 7 patients underwent only OR. None required additional femoral osteotomy as per Pre-op MRI parameters (FA <50°) and intra operative assessment for stability and pressure on femoral head.

Pre-operatively and post-operatively, we assessed the limb length discrepancy, any limp or pain on walking, the Trendelenburg test, and the telescopy test. After six months of follow-up, on the clinical evaluation, we found 12 hips in Grade 1 and 3 hips (all were OR-only group cases) in Grade 2 according to Modified McKay's criteria. No complications like wound infections, hip re-dislocation, or AVN changes were noted.

## Discussion

The treatment of DDH in walking age is open reduction coupled with or without acetabular and/or femoral osteotomy^19^. In children belonging to Low- and middle-income countries (LMIC), the parents usually bring the DDH child late, only after noticing a limp when the child starts to walk, and by then, the hip becomes irreducible. They usually present with various complaints like toe walking, limb length discrepancy, Trendelenburg's gait, postural scoliosis, etc., and the treatment options narrow down to only open reduction^20^. It is postulated that the pathology primarily lies in the acetabulum, and the changes in the femur are secondary^21^.

The goals of treatment in developmental dysplasia of the hip (DDH) are obtaining and maintaining a concentric hip reduction and avoiding growth disturbance. There is a debate among practitioners regarding when to perform acetabular osteotomy or femoral varus derotational osteotomy (VDRO)^22^.

Ultrasound is usefully recommended up to six months of age before the femoral head ossification centre appears (four to six months), and after that, radiographs are taken to diagnose DDH^19^. MRI has the advantage of being radiation-free. Its ability to delineate cartilaginous and soft tissue structure (multiplanar capability) appears to be an ideal modality for assessing pre-operative parameters and post-operative reduction parameters in the relatively unossified skeleton. It can pick up any early post-operative avascular necrosis changes, too^23^. The present study aims to compare MRI-based parameters with the intra-operative position of stability towards achieving the goal of concentric reduction and compare pre-operative MRI parameters with post-operative ones.

In this study, the right side alone was involved in 53% of cases. All of our patients were females. These demographic findings are consistent with previous literature regarding female sex predominance^24^. The mean age of OR with Salter’s group (37.00±7.94 months) is higher than the OR-only (25.00±3.51 months) group.

In our study, the mean FA on the involved side was 35.27°, while the mean FA on the normal was 36.6°. The range on the involved side was 11-44°, and on the normal side, it was 2250°. However, the difference of mean FA between the involved and normal side was statistically not significant (p=0.67). Our study’s statistical insignificance of FA was comparable to Sarban *et al* and Mootha *et al*^25,26^. Sarban *et al* found a mean FA of 32.90±6.41° in the dislocated group and 30.72±6.10° in the normal group^25^. Mootha *et al* found the mean FA to be 23.18±5.08° on the dislocated side ranging from 13 to 29°, while on the normal side, it was 21.36±4.57°, ranging from 14 to 28°^26^.

Based upon pre-op MRI FA calculations, no hip required femoral derotational osteotomy, and the same results were found intra-operatively that no hip required the position of internal rotation in addition to abduction for maximum stability. After six months post-operative MRI value of femoral anteversion was comparable in both control and involved hip as we found pre-operatively.

Regarding acetabular anteversion, the mean AA on the involved side was 23.78°, while the on the normal side was 22.78°. There was a statistically insignificant difference (p=0.62) between the normal and involved sides. The mean post-op AA on the normal side was 18.25° and on the tested side was 17.88° which was also statistically insignificant. Sarban *et al*^25^ found the acetabular anteversion to be 13.4±2.8° (mean±SD) in normal hips and 19.8±2.5° in completely dislocated hips. There was a statistically significant difference between the two groups in their study. In OR with Salter's and OR-only group cases, we also found insignificant results. The paired difference in the mean value of AA of OR with Salter's group cases was 1.41 and 0.83 in normal and involved side, respectively, whereas in OR-only group cases, it was 1.28 and 1.72.

The mean cartilaginous acetabular index on the involved side was found to be 42.80°, while the mean index on the normal side was 22.13°. The difference between the normal and involved sides was statistically significant (p=0.00). Preoperative MRI predicted a need for Salter osteotomy in 14 hips. But based on the intra-operative position of stability, we did Salter's innominate osteotomy in eight hips and open reduction only in seven hips. We were able to achieve a concentric reduction in all of them. The difference in the ability of pre-operative MRI to predict an osteotomy and intra-operative position of stability was significant and contemplating osteotomy.

As we assess, all cases in which Salter's osteotomy was done had good results clinically and radiologically. Mean pre-op CAI in OR with Salter's osteotomy group was 22.50° on the normal side, and on the involved side, it was 43.01°. Post-op AI in the same group was 19.21° on the normal side, and the involved side was 20.90°. The paired difference in mean CAI pre-op was 20.51, and post-op was 1.70. Modified McKay's criteria used to evaluate all Salter osteotomy cases, and we found all hip in the Grade 1 category.

In seven cases, intra-operatively, we found maximum stability in a neutral position, we did only open reduction, but according to pre-op MRI, six out of seven required Salter's osteotomies. Mean pre-op CAI in OR-only cases on the normal side was 21.15°, and on the involved side was 42.50°. Post-op AI in the same group was 19.01° on the normal side, and on the involved side, it was 25.0. The paired difference in mean AI pre-op was 20.857, and post-op was 5.671.

According to Modified McKay’s criteria, in the OR-only group, we found four hips in grade 1 and 3 hips in the grade 2 category. We also found insignificant results in the paired difference in mean AI of OR with Salter and OR-only group cases.

To the best of our knowledge, we did not find any study on anterior and posterior sector angle (ASA and PSA) of the acetabulum, which showed anterior and posterior coverage of femur head. In hip dysplasia, the most common finding is inadequate anterior coverage, with decreased ASA^27^. Postoperatively we compared ASA and PSA with the normal side. Overall, the mean ASA on the normal side was 61.45° and on the involved side was 58.07°, while PSA was 79.26° on the normal side and 68.0° on the involved side. The mean postoperative ASA is more (although not statistically significant) in OR with Salter’s group rather than OR-only group and it shows Salter’s osteotomy provided increased anterior coverage.

The percentage of femoral head coverage was 0 in pre-op cases as the head was dislocated. Post-operatively we compared PFHA with the normal side. Mean PFHA on the normal side was 91%, while on the involved side was 79%. Further, in OR with Salter's group, all cases have PFHA greater than 75% while three cases of OR only group have PFHA less than 75%. Long follow-up of these cases will be required to assess the evolution of coverage.

Finally, we infer that the decision to make acetabular re-directional osteotomy is not based on pre-operative MRI parameters but according to the intra-operative stability assessment. Arslan *et al* also concluded the same that the decision to make pelvic osteotomy is not based on the age (more than or less than 18 months old) of the child but based on intra-operative stability^28^.

We felt a need for a comprehensive morphometric study on Indian paediatric hips during the research, as much of the literature is on the western and Chinese populations^6,29^. The well-defined range of parameters would augment decision-making in even bilateral cases.

A word of caution is also justified against over-dependence on MRI at present. More sets of parameters may be needed to be studied to make MRI even more reliable. As our study is of shorter duration follow-up, we feel longer follow-up will yield much more comprehensive results. Another set of the area which needs attention is cost-effectiveness and duration of MRI study period. Although MRI is much safer than CT regarding radiation exposure, affordability for both pre-op and post-op MRI is limited and the need for sedation is an issue.

## Conclusion

Although pre-operative MRI helps assess the acetabular and femoral parameters, it is not dynamic. At a given point of time, only two-dimensional (2D) parameters are taken into account for assessment and based upon which pre-operative planning is done. But an intra-operative stability check is a dynamic procedure that considers the three-dimensional (3D) morphology and the best possible inter-relation between the articular surface of the femoral head and acetabulum. Intra-operatively, it helps in deciding when to do an additional acetabular re-directional osteotomy to maintain a stable and concentric reduction. So, routine pre-operative MRI is not advised for surgical planning of every DDH case and may be reserved for revision or complex cases.
